# Incipient Fault Detection in a Hydraulic System Using Canonical Variable Analysis Combined with Adaptive Kernel Density Estimation

**DOI:** 10.3390/s23198096

**Published:** 2023-09-26

**Authors:** Jinxin Wang, Shenglei Zhao, Enyuan Wang, Jiyun Zhao, Xiaofei Liu, Zhonghui Li

**Affiliations:** 1 School of Safety Engineering, China University of Mining and Technology, Xuzhou 221116, China; wangjinxin@cumt.edu.cn (J.W.); shengleizhao@cumt.edu.cn (S.Z.); liuxiaofei@cumt.edu.cn (X.L.); lizhonghui@cumt.edu.cn (Z.L.); 2 School of Mechanical and Electrical Engineering, China University of Mining and Technology, Xuzhou 221116, China; jyzhao@cumt.edu.cn

**Keywords:** hydraulic system, condition monitoring, fault detection, canonical variable analysis, adaptive kernel density estimation

## Abstract

Incipient fault detection in a hydraulic system is a challenge in the condition monitoring community. Existing research mainly monitors abnormal working conditions in hydraulic systems by separately detecting the key working parameter, which often causes a high miss warning rate for incipient faults due to the oversight of parameter dependence. A principal component analysis provides an effective method for incipient fault detection by taking the correlation of multiple parameters into consideration, but this technique assumes the systems are Gaussian-distributed, making it invalid for a dynamic non-Gaussian system. In this paper, we combine a canonical variable analysis (CVA) and adaptive kernel density estimation (AKDE) for the early fault detection of nonlinear dynamic hydraulic systems. The collected hydraulic system data set was used to construct the typical variable space, and the state space and residual space are divided to represent the characteristics of different correlations between the two variables, which are quantitatively described using Hotelling’s *T*^2^ and *Q*. In order to investigate the proper upper control limits, AKDE was utilised to estimate the underlying probability density functions of *T*^2^ and *Q* by taking the nonlinearity of the hydraulic system variables into consideration. The advantages of the proposed approach for incipient fault detection are illustrated via a marine power plant lubrication system.

## 1. Introduction

The influence of hydraulic systems, ranging from oil production platforms and truck-mounted drilling rigs to powered supports in coal mining, on various engineering equipment in modern society has been increasing [1]. In practice, a hydraulic system often works with heavy loads and varying working conditions, and thus, various faults may occur during its life cycle [2]. The timely detection of the operation behaviour as well as the key working parameters is crucial to ensure safe operation and achieve the desired performance of mechanical systems.

One can distinguish between three types of hydraulic system faults according to time evolution: abrupt, intermittent, and incipient [3,4]. Currently, most existing research focuses on monitoring abrupt and intermittent faults in hydraulic systems [5,6,7,8,9,10] and the detection of incipient fault; however, the findings are limited. An incipient fault in a hydraulic system is characterised by a slow degradation of system performance. If left undetected, incipient faults will keep increasing in severity and lead to catastrophic system failure as a consequence. Although necessary, the detection of incipient faults in hydraulic systems is a great challenge because the observed system parameters usually do not show notable deviations at the incipient fault stage [11], which makes existing independent univariate-based condition monitoring techniques (typically Pauta criterion-based methods) invalid for incipient fault detection. Some studies are devoted to applying advanced signal processing and deep learning approaches for incipient fault detection in the hydraulic systems of critical equipment. For example, Arunthavanathan et al. proposed a multi-step multivariate CNN-LSTM deep neural network interface with a one-class support vector machine for the process of incipient fault detection. In their study, early fault detection is regarded as a time-dependent sequence learning problem, and fault symptoms are forecast using LSTM from the previous data pattern to monitor an early fault [12]. Deng et al. proposed an online fault detection approach by combining a space–time compressed matrix and Naive Bayes, which largely reduces the learning complexity and ensures early fault detection [13]. Cheded et al. proposed a novel integrated framework for incipient fault detection in terms of process safety. Multiple deep learning approaches are integrated in their research [14]. Although approaches to incipient fault detection in novel hydraulic systems continue to emerge [15,16,17,18,19,20], the working condition of a hydraulic system is essentially still evaluated via the change magnitude of feature values, which has the same disadvantages as those described above and is found to be unsatisfactory in practice.

Risk-based fault detection and diagnosis have successfully developed in recent years, largely improving the working safety of hydraulic systems. Among all process fault detection methodologies, multivariate statistical process monitoring (MSPM) is an advanced data-driven condition monitoring approach [21]. Different from the independent univariate-based approach described above, this technique monitors a fault by taking both the change in parameter/feature values as well as the change in correlations of various parameters/features into consideration [22,23,24,25,26,27,28]. Our previous research introduced the MSPM for incipient fault detection in hydraulic systems [29], and an improved ability for incipient fault detection was achieved compared with the independent univariate-based approach. The multi-parameter data of the state equation can realise the early fault monitoring and output tracking control of the system, and its diagnosis ability is strong, but it is difficult for it to effectively face the complex dynamic non-Gaussian system [30]. Existing MSPM monitoring techniques like principal component analysis (PCA) and partial least square (PLS) can be used to construct static fault detection models by assuming the observations are time-independent and follow a Gaussian distribution [31,32,33,34,35]. A hydraulic system, in practice, is a time-varying system, and past states usually have strong effects on the future working states (i.e., parameters are strongly auto-correlated). Moreover, observations often show non-Gaussian characteristics due to the load disturbance and system parameter uncertainties. The assumptions of time independence and normality fail to capture the dynamic characteristics of a hydraulic system, and thus, PCA and PLS techniques are not suitable for nonlinear dynamic system fault detection. Some research extends PCA and PLS applications to dynamic systems, i.e., DPCA and DPLS [36,37]. Nevertheless, research shows that the principal components extracted by DPCA and DPLS are not necessarily for minimal dynamic representations, and the detection of abnormalities is more complicated when lagged variables are involved [38,39].

Canonical variate analysis (CVA) is a state-space-based system and dynamic characteristic analysis approach [40]. The state subspace and residual subspace of an engineering system are constructed according to the correlations between past and future system behaviour using CVA, and the performance shifts can be effectively detected by checking how well future system behaviour is predictable from a past state [41,42,43,44,45]. However, existing MSPM technology is mostly used in chemical industry condition monitoring [46]; the application on mechanical hydraulic systems is found limited. Moreover, existing MSPM techniques determine upper control limits (UCLs) of multivariate statistical metrics based on Gaussian assumption. For most hydraulic systems, although the system disturbance and measurement noise are Gaussian, the latent or state variables are non-Gaussian due to the system nonlinearity. In such a case, the UCLs derived using the Gaussian assumption are unable to correctly identify the underlying system faults [47,48,49,50,51].

In this paper, a novel hydraulic systems incipient fault detection approach is proposed using CVA and a new extension of KDE, i.e., Adaptive KDE (AKDE), by overcoming the disadvantages aforementioned. The state space and residual space of hydraulic systems are firstly constructed using CVA by modelling the time-varying characteristics of system variables. Two multivariate statistics, $T^{2}$ and Q, are then introduced as monitoring metrics to perceive the system abnormal working condition. In order to investigate the proper upper control limits, AKDE is utilised to estimate the underlying probability density functions of $T^{2}$ and Q by taking the nonlinearity of the hydraulic system latent or state variables into consideration. Compared with existing research, the main contributions of the paper are summarised in the following: (i) a novel CVA-based methodology is proposed to improve the incipient fault detection ability of a dynamic hydraulic system; (ii) two novel multivariate statistical variables, i.e., $T^{2}$ and Q, are constructed as metrics for hydraulic system condition monitoring; and (iii) a procedure for assigning the UCLs of $T^{2}$ and Q is given by estimating the PDFs of multivariate statistical metrics using AKDE.

The rest of this paper is organised as follows: Section 2 briefly introduces the principle of MSPM for hydraulic systems incipient faults detection. Section 3 describes the CVA model of a hydraulic system. Section 4 explains monitoring metrics and their UCLs derived through AKDEs. A case study is given in Section 5. Finally, Section 6 summarises the paper.

## 2. MSPM for Incipient Faults Detection Section

The condition monitoring of an engineering system is usually achieved via separately monitoring the key working parameters or features which define the performance of the system to some extent. This technique has limited ability to detect small performance shifts, which are the basic characteristics of incipient faults. The difficulties of this technique for detecting incipient faults are that the system parameters/features are not independent from each other, and any one of them is not adequate to define the work performance of a system. Therefore, the fault detection of a system has multivariate properties, which means the performance of a system should be defined by the correct simultaneous values of all observed parameters.

**Figure 1:**
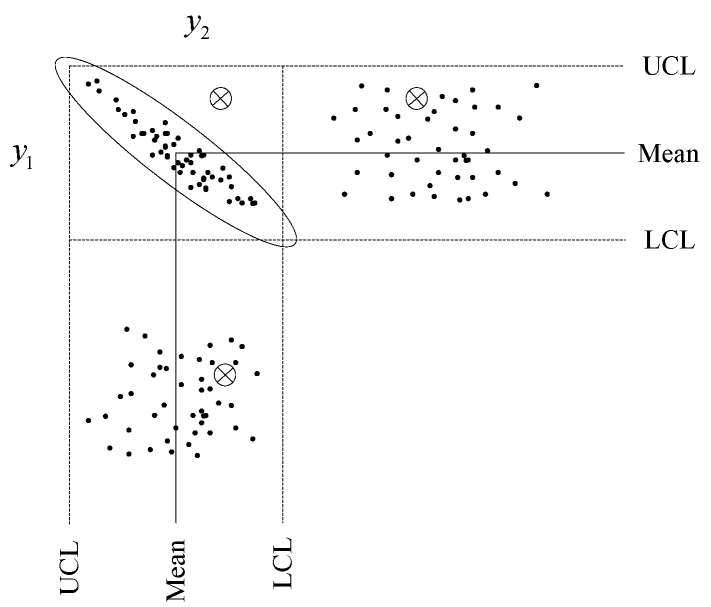
Multivariate property of incipient faults detection.

The principle and advantages with using MSPM techniques detecting incipient faults can be illustrated via Figure 1, where $y_{1}$ and $y_{2}$ are two working parameters of a system and follow a multivariate normal distribution with a correlation relationship; the ellipse in Figure 1 depicts the normal fluctuation range of a system in control; dark spots represent a set of observations while the asterisk means the faulty condition; the dashed lines in Figure 1 represent the UCLs and lower control limits (LCLs), respectively. By inspection of each individual observation, the system is clearly working in a good condition, and none of the observations gives any indication of a problem. The fault can only be pinpointed by the correlation of multivariate $y_{1}$ and $y_{2}$, where the asterisk is outside of the confidence region. The interdependent characteristics and confidence regions of multiple system parameters are determined via state-space-based methods.

This example clearly shows the difficulties of independent univariate-based approach for incipient fault detection and also shows the advantages of the MSPM approach on this problem. In the following parts, we show the CVA model for hydraulic systems incipient fault detection.

## 3. Canonical Variable Analysis of Hydraulic Systems

Canonical variable analysis establishes a state-space model of a dynamic system by finding the maximum correlation between past and future variables in the normal condition. Let $x_{k}\in {\mathbb{R}}^{n}$ and $u_{k}\in {\mathbb{R}}^{l}$ be the state and input vectors, respectively, and $y_{k}\in {\mathbb{R}}^{m}$ be the observation vector; then, a nonlinear dynamic hydraulic system can be represented in a vector autoregressive moving average time-series model with exogenous inputs, i.e.,

(1) $$\left\{ \begin{array}{l} x_{k+1}=f\left(x_{k}\right)+h\left(u_{k}\right)+p_{k}\\ y_{k}=g\left(x_{k}\right)+w\left(u_{k}\right)+v_{k} \end{array}\right.$$

where f(⋅), h(⋅), g(⋅) and w(⋅) are unknown nonlinear functions of the system; while $p_{k}$ and $v_{k}$ are system disturbance and measurement noise.

The input variables of a system are constant at a stable normal operating point. Therefore, the nonlinear dynamic system can be described as a stochastic state-space model, and Equation (1) is then converted into a more computable form as

(2) $$\left\{ \begin{array}{l} x_{k+1}=Ax_{k}+\varepsilon_{k}\\ y_{k}=Cx_{k}+\eta_{k} \end{array}\right.$$

where A and C are coefficient matrices; $\varepsilon_{k}$ and $\eta_{k}$ are the modelling errors partially arising from the system nonlinearity not being modelled, as well as the system disturbance $p_{k}$ and measurement noise $v_{k}$. In spite of the vectors, $p_{k}$ and $v_{k}$ may be Gaussian distributed, $\varepsilon_{k}$ and $\eta_{k}$ are general non-Gaussian due to the existence of nonlinearity. The dynamic and non-Gaussian characteristics of a hydraulic system are taken into consideration using the CVA approach and AKDE for incipient faults detection, which are the main contributions of this work from other reported MSPM approaches.

Suppose $y_{k}$ is a set of observations at a certain time point k; then, the past and future measurement vectors scaled to a zero mean and unit variance for each time point k are given as

(3) $$y_{p,k}=\left[\begin{array}{c} y_{k-1}\\ y_{k-2}\\ \vdots \\ y_{k-q} \end{array}\right]\in {\mathbb{R}}^{mq}$$

(4) $$y_{f,k}=\left[\begin{array}{c} y_{k}\\ y_{k+1}\\ \vdots \\ y_{k+q-1} \end{array}\right]\in {\mathbb{R}}^{mq}$$

where q represents the quantity of lags in the time windows and mq depicts the lengths of past and future measurement vectors $y_{p,k}$ and $y_{f,k}$. The variable q is determined according to the auto-correlation of system parameters.

As for a sample data set with N observations, in total, M=N−2q+1 training sets can be obtained with time windows shifting. For all time points k∈[p+1,p+M], the past and future Hankel matrices can be formed by appending columnwise measurement vectors $y_{p,k}$ and $y_{f,k}$

(5) $$Y_{p}=\left[y_{p,q+1}\;y_{p,q+2}\;\ldots \;y_{p,q+M}\right]\in {\mathbb{R}}^{mq\times M}$$

(6) $$Y_{f}=\left[y_{f,q+1}\;y_{f,q+2}\;\ldots \;y_{f,q+M}\right]\in {\mathbb{R}}^{mq\times M}$$

The CVA constructs the state space of a system by finding the linear combinations of past and future variables with maximum correlation. Let $a^{T}\left(y_{f,k}\right)$ and $b^{T}\left(y_{p,k}\right)$ be the coefficient vectors of the linear combinations; then, the correlation of past and future variables can be written as

(7) $$\rho_{fp}\left(a,b\right)=\frac{a^{T}\sum_{fp}b}{{\left(a^{T}\sum_{ff}a\right)}^{1/2}{\left(b^{T}\sum_{pp}b\right)}^{1/2}}$$

where $\sum_{pp}\cdot$, $\sum_{ff}\cdot$ and $\sum_{fp}\cdot$ are the covariance and cross-covariance matrices, and these can be given as

(8) $$\sum_{pp}\cdot =\frac{1}{M-1}\sum_{k=q+1}^{q+M}y_{p,k}y_{p,k}^{T}=\frac{1}{M-1}Y_{p}Y_{p}^{T}$$

(9) $$\sum_{ff}\cdot =\frac{1}{M-1}\sum_{k=q+1}^{q+M}y_{f,k}y_{f,k}^{T}=\frac{1}{M-1}Y_{f}Y_{f}^{T}$$

(10) $$\sum_{fp}\cdot =\frac{1}{M-1}\sum_{k=q+1}^{q+M}y_{f,k}y_{p,k}^{T}=\frac{1}{M-1}Y_{f}Y_{p}^{T}$$

Let $u=\sum_{ff}^{-1/2}a$ and $v=\sum_{pp}^{-1/2}b$ for simplification be an expression of Equation (7). The optimisation problem is then converted into finding vectors u and v, which maximise Equation (7), i.e., $\sigma =\underset{a,b}{\max}\rho_{fp}(a,b)$. It has been proven that the optimal solutions of u and v can be pursued via the singular value decomposition (SVD) of a Hankel matrix $H=\sum_{ff}^{-1/2}\sum_{fp}\sum_{pp}^{-1/2}$, i.e.,

(11) $$H=\sum_{ff}^{-1/2}\sum_{fp}\sum_{pp}^{-1/2}=UDV^{T}$$

where U and V are the left and right singular vectors of H, respectively; while D is a diagonal matrix of singular values (SVs), which is sorted in descending order, $\sigma_{1}\geq \sigma_{2}\geq \cdots \geq \sigma_{r}$, i.e.,

(12) $$U=\left[u_{1}\;u_{2}\;\cdots \;u_{r}\right]\in {\mathbb{R}}^{mq\times r}$$

(13) $$V=\left[v_{1}\;v_{2}\;\cdots \;v_{r}\right]\in {\mathbb{R}}^{mq\times r}$$

(14) $$D=\left[\begin{array}{cccc} \sigma_{1} & 0 & \cdots & 0\\ 0 & \sigma_{2} & \cdots & 0\\ \vdots & \vdots & \ddots & \vdots \\ 0 & 0 & \cdots & \sigma_{r} \end{array}\right]\in {\mathbb{R}}^{r\times r}$$

where r is the rank of the Hankel matrix H. The singular vectors $u_{i}$ and $v_{i}$ correspond to the coefficient of the system variables’ linear combinations, and the correlation of the new combined variables, i.e., canonical variates (CVs) is $\sigma_{i}$, which is the SVs corresponding to $u_{i}$ and $v_{i}$. The canonical variates space of the system measurement is formed as

(15) $$z_{k}=\left[\begin{array}{c} b_{1}^{T}\\ b_{2}^{T}\\ \vdots \\ b_{r}^{T} \end{array}\right]y_{p,k}=\left[\begin{array}{c} v_{1}^{T}\\ v_{2}^{T}\\ \vdots \\ v_{r}^{T} \end{array}\right]\sum_{pp}^{-1/2}y_{p,k}=V^{T}\sum_{pp}^{-1/2}y_{p,k}$$

The r-dimensional canonical variates space is composed of state space and residual space. The state space is spanned first by n CVs, which explains most of the characteristics of system measurements, while the residual space is spanned by the remaining (r−n) CVs, which mainly represent modelling the residual information of CVA. The state space $x_{k}$ and residual space $e_{k}$ at time point k can be represented as

(16) $$x_{k}=J_{x}y_{p}{{}_{,}}_{k}=V_{x}^{T}\sum_{pp}^{-1/2}y_{p}{{}_{,}}_{k}$$

(17) $$e_{k}=Fy_{p}{{}_{,}}_{k}=\left(I-V_{x}V_{x}^{T}\right)\sum_{pp}^{-1/2}y_{p}{{}_{,}}_{k}$$

where $J_{x}$ and F depict the projection matrices of state space and residual space, respectively; $V_{n}$ is a reduced matrix formed by the first n columns of V.

Hotelling’s $T^{2}$ and Q indexes are used to test the measures of the state of the principal component space and the residual space, respectively, covering the two different characteristics of the collected data. Two multivariate statistics, $T^{2}$ and Q, are constructed, respectively, to evaluate the variation of state variables of modelling errors. The estimations of $T^{2}$ and Q metrics generated at time point k are given as

(18) $$T_{k}^{2}={\left(x_{k}-\tau \right)}^{T}S^{-1}\left(x_{k}-\tau \right)=x_{k}^{T}x_{k}$$

(19) $$Q_{k}=e_{k}^{T}e_{k}$$

where τ is the desired mean of x, and S is the estimated covariance. The second equation in Equation (18) holds, since the measurements are normalised to a zero mean and unit variance, i.e., τ=0 and S=I.

A hydraulic system modelled in Equations (1) and (2) can be monitored according to the $T^{2}$ and Q plots against time k. An abnormality is reported when the multivariate statistics fluctuate exceeding their upper control limits $T_{UCL}^{2}$ and $Q_{UCL}$. The way to assign suitable $T_{UCL}^{2}$ and $Q_{UCL}$ values is explained in the next section.

## 4. Upper Control Limits Determination Using AKDE

For a hydraulic system described in Equation (2), if we ignore the system nonlinearity and only take system disturbance $p_{k}$ and measurement noise $v_{k}$ into consideration, the measurements will follow Gaussian distributed, and then, we have $CT^{2}∼F\left(n,M-n\right)$ where C=M(M−n)/(M−1)(M+1)n. Given a confidence level α, the upper control limit $T_{UCL}^{2}$ can be derived as

(20) $$T_{UCL}^{2}=\frac{n\left(M-1\right)\left(M+1\right)}{M\left(M-n\right)}F_{n,M-n}\left(\alpha \right)$$

where $F_{n,M-n}(\alpha )$ represents the critical value of F distribution with freedoms of n and (M−n) at a confidence level α.

The UCL of Q metric can be estimated based on Gaussian distribution as

(21) QUCL(α)=θ1(h0cα2θ2θ1+θ2h0(h0−1)θ12+1)1h0

where $c_{\alpha }$ is the critical value of Gaussian distribution at a confidence level α; $\theta_{i}\left(i=1,2,3\right)$ and $h_{0}$ are calculated as

(22) $$\left\{ \begin{array}{l} \theta_{i}=\sum_{j=n+1}^{r}\lambda_{j}^{i}\\ h_{0}=1-2\theta_{1}\theta_{2}/3\theta_{2}^{2} \end{array}\right.$$

where $\lambda_{j}$ is the covariance of system measurements, $\lambda_{j}=\sum_{pp}^{-1/2}y_{p}{{}_{,}}_{k}$.

The upper control limits in Equations (20) and (21) are derived under the assumptions that the measurements are Gaussian, and the nonlinearity of the hydraulic system behaviour is not directly accounted for. Hence, this assumption is valid for a hydraulic system in practice and inevitably leads to misdiagnosis. This section presents a novel AKDE-based upper control limits assignation approach by estimating the actual probability density functions of $T^{2}$ and Q metrics for a hydraulic system.

Since the $T^{2}$ and Q metrics can be viewed as random variables, the estimation of PDFs can be explained using just one general variate X. Suppose $\left\{x_{1},x_{2},\cdots ,x_{g}\right\}$ are M independent samples of X; then, the estimated PDF using AKDE can be formed as follows given a kernel function K(⋅).

(23) $$\hat{f}\left(x\right)=\frac{1}{Mh}\sum_{k=1}^{M}K\left(\frac{x-x_{k}}{h}\right)$$

where h is the bandwidth.

The bandwidth h has a direct influence on the estimation accuracy. An improper h would make the estimated PDF deviate from the real one, and it would further decrease the fault detection rate. The AKDE overcomes the disadvantages of a fixed-width KDE by utilising an adaptive bandwidth which assigns a larger width in regions of a lower probability density and a smaller width otherwise. An initial bandwidth $h_{0}$ is firstly set as Equation (24) according to the principle of minimising the mean integrated square error.

(24) h0=(4σ^53M)15

where $\hat{\sigma }$ is the standard deviation of X.

A pilot PDF estimation ${\hat{f}}_{p}(x)$ is then produced using fixed-width KDE. The local bandwidth factor $\tau_{i}$ is calculated to evaluate the adaptation of initial bandwidth $h_{0}$ in regions with different probability density

(25) $$\tau_{i}={\left\{{\left[\prod_{j=1}^{M}{\hat{f}}_{p}\left(x_{j}\right)\right]}^{\frac{1}{M}}/{\hat{f}}_{p}\left(x_{i}\right)\right\}}^{\xi }$$

where ξ is the sensitivity factor.

The initial bandwidth $h_{0}$ in different regions is modified using $\tau_{i}$ to adapt the characteristic of data distribution. The estimated PDF of X using AKDE is defined as

(26) $$\hat{f}\left(x\right)=\frac{1}{M}\sum_{k=1}^{M}\frac{1}{\tau_{i}h_{0}}K\left(\frac{x-x_{k}}{\tau_{i}h_{0}}\right)$$

The procedure above shows the estimation of $T^{2}$ and Q probability density functions. The upper control limits of $T^{2}$ and Q metrics at a confidence level α can be derived according to the estimated PDFs.

(27) $$\left\{ \begin{array}{l} \int_{-\infty }^{T_{UCL}^{2}}\hat{f}\left(T^{2}\right)dT^{2}=\alpha \\ \int_{-\infty }^{Q_{UCL}}\hat{f}\left(Q\right)dQ=\alpha \end{array}\right.$$

The $T^{2}$ and Q metrics are combined for hydraulic system condition monitoring. The system performance is thought to deviate from normal conditions if the following rule holds.

(28) $$\left(T^{2}>T_{UCL}^{2}\right)\vee \left(Q>Q_{UCL}\right)$$

where ∨ represents a logical OR operation.

## 5. Case Study

### 5.1. Hydraulic Lubrication System of a Large Marine Power Plan

This section takes the hydraulic lubrication system of a large marine power plant as an example to illustrate the proposed approach, given that the working condition of the lubrication system is critical to a large marine power plant, and the working pressures and oil temperatures are key parameters for lubrication system control.

**Figure 2:**
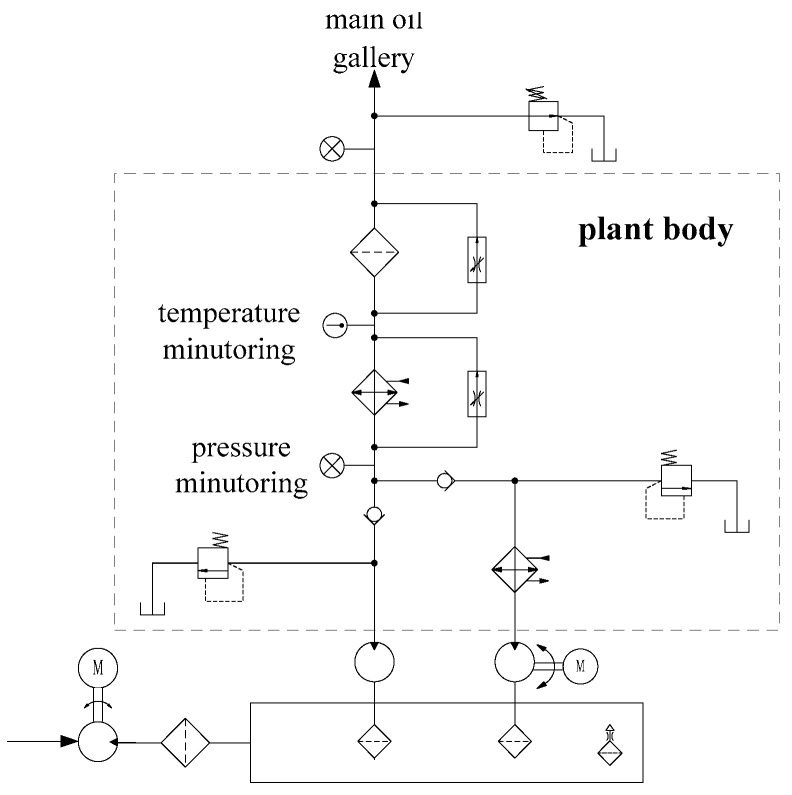
Forced-feed hydraulic lubrication system of a marine power plant.

The hydraulic lubrication system continuously provides clean, cool lubrication and hydraulic fluid to the marine power system. Figure 2 shows the process diagram of a typical marine power plant forced-feed lubrication system, which is composed of several components including a gear pump, motor pump, cooler, filter, check valves, pressure-regulating valves and pressure-relief valves. The working process of the lubrication system is divided into two stages: a pre-lubricating stage and regular lubricating stage. In the pre-lubricating stage, the oil is drawn via a motor pump to provide a marine power plant with the lubrication and hydraulic fluid before start-up, and the controlled heat exchanger is used to heat the oil to a proper temperature for a cold boot. In the regular lubricating stage, the motor pump is shut down, and oil is drawn via a gear pump which is driven directly by the crankshaft through a transmission mechanism. Pressure relief valves are installed behind oil pumps to prevent the system components being damaged by over-high pressure.

From the oil pump, the oil enters the cooler and filter successively where the oil temperature is adjusted to a desired value and the impurities are removed from the lubricating oil. The oil cooler is connected with a heat control valve in parallel, and the valve opens to make the oil bypass the cooler when the temperature is too low. A bypass valve is configured in the pipeline to guarantee the oil supply. When the filter is blocked, the differential pressure at both ends of the filter will exert sufficient force against the compression spring to open the valve, and the oil would pass through the valve for oil supply in an emergency. Oil then enters into the power plant with a certain pressure and temperature through the main oil gallery. A pressure-regulating valve is installed in the main oil gallery to control the oil pressure for the desired range. The oil out of the plant flows back to the sump for the next cycle.

### 5.2. Incipient Fault Detection Using CVA Combined with AKDE

Three common gradual faults of a marine power plant hydraulic lubrication system, i.e., pump failure $f_{1}$, pipe leakage $f_{2}$ and filter blockage $f_{3}$, are considered in this paper, given the occurrence frequency and seriousness of the failures. The performance of the hydraulic lubrication system is described via four key working parameters: oil pressure after pump $s_{1}$, oil pressure before filter $s_{2}$, oil pressure after filter $s_{3}$ and oil temperature after filter $s_{4}$. The working parameters are collected via a National Instruments PCI-6225 data acquisition system. The median filter is used to preprocess the acquired data and reduce the noise of the thermodynamic and mechanical measurement signals [52]. The marine power plant is working at three typical working conditions, i.e., 25%, 50%, and 75% full loads at 1800 r/min. Table 1 shows the fault categories of the source datasets. The training dataset and test dataset are constructed using the collected system’s working parameters. The training dataset is composed of normal data which is used to determine the control limits of system parameters, while the test datasets are composed of both normal data and fault data to evaluate the performance of a fault detection approach.

**Figure 3:**
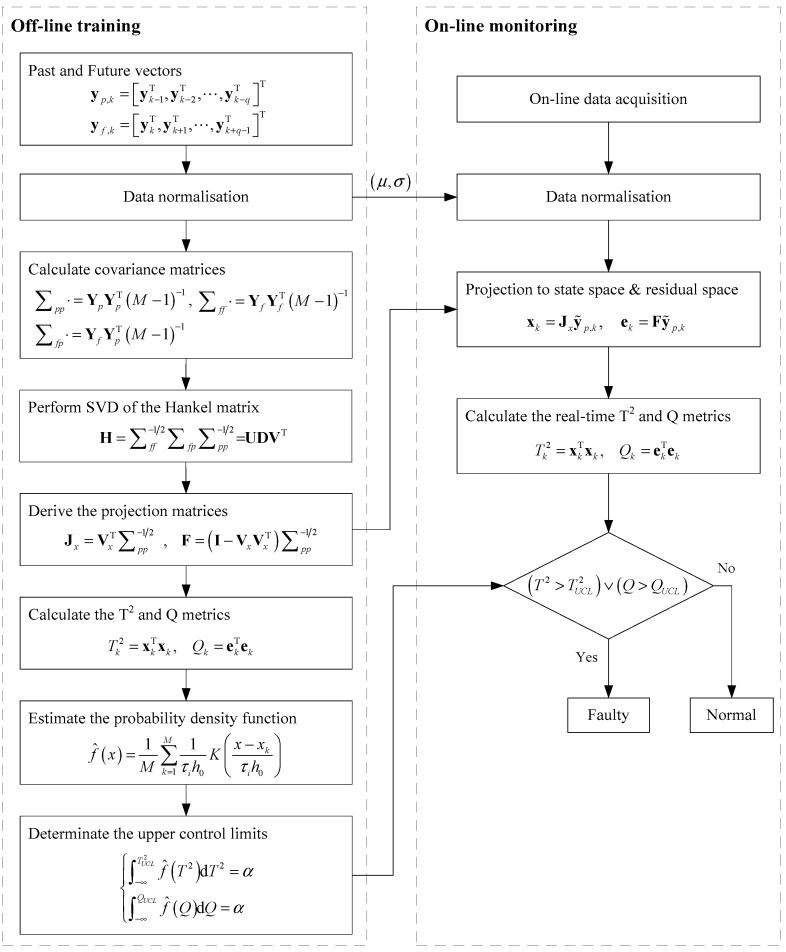
Incipient fault detection procedure using CVA with AKDE.

The working condition of the hydraulic lubrication system is monitored using Pauta criterion, PLS, a PCA-based multivariate statistical approach and the proposed CVA with an AKDE approach, respectively. The Pauta criterion is the most widely used univariate statistics-based fault detection method in practice, while PLS and PCA are the two most classical MSPM condition monitoring approaches and have been successfully used in processing monitoring. The comparisons with the two approaches aim to reveal the advantages of MSPM for incipient fault detection and to illustrate the improvements regarding fault detection performance by taking system time-varying characteristics into consideration using the proposed CVA with AKDE. The incipient fault detection technical procedure using CVA with AKDE is shown in Figure 3, which is divided into two steps. The off-line training step generates the state and residual subspaces and estimates the PDFs of $T^{2}$ and Q metrics to assign the UCL for each system’s working condition via learning the collected healthy datasets. The online monitoring step deduces the $T^{2}$ and Q metrics according to the real-time detected system parameters and compares with the upper control limits, respectively, to monitor the healthy condition of the hydraulic system. The monitoring performance of each approach is evaluated according to the fault detection rate (FDR) $r_{d}$, which is defined as follows, in which L represents the total number of fault samples while $n_{c}$ depicts the fault sample number successfully identified.

(29) $$r_{d}=\frac{n_{c}}{L}\times 100\%$$

**Figure 4:**
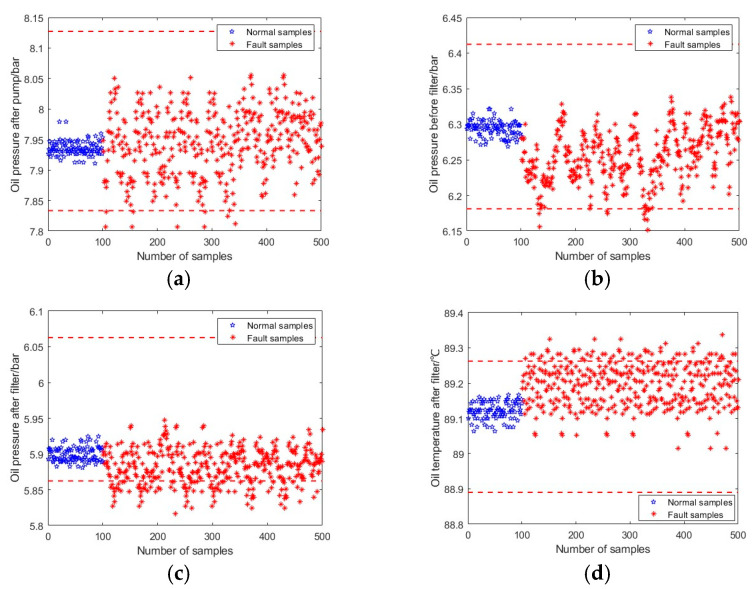
Fault detection of pump failure using Pauta criterion (50% full load, 1800 rpm). (**a**) Oil pressure after pump. (**b**) Oil pressure before filter. (**c**) Oil pressure after filter. (**d**) Oil temperature after filter.

This section firstly takes the pump failure $f_{1}$ at 50% full load, 1800 r/min as an example to illustrate the fault detection process using the three approaches above. Figure 4 shows the fault detection results using the Pauta criterion, in which the blue spots represent the data samples of healthy condition, whereas the red spots depict the fault data samples. The red lines represent the UCLs and LCLs of the corresponding system parameters, which are determined based on the mathematical expectations and standard deviations of system parameters in healthy conditions. A fault is reported if any of the system parameters are exceeding their corresponding control limits. According to the fault detection results presented in Figure 4, only a small portion of parameter samples exceed their thresholds and are recognised as fault samples, whereas most of the fault samples failed to report because all parameters still fluctuate within their control limits. The FDR of the Pauta criterion is calculated as 36%, which shows that the Pauta criterion are unsatisfied for hydraulic system incipient fault detection. In addition, the small deviations of system parameters also present the features of incipient faults.

**Figure 5:**
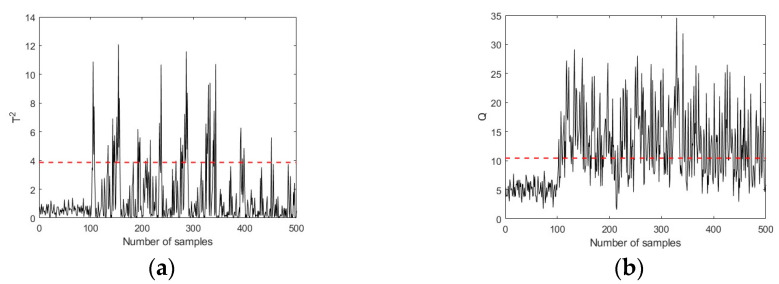
Pump failure detection using PLS (50% full load, 1800 rpm). (**a**) Hotelling’s $T^{2}$ in PLS. (**b**) Hotelling’s Q in PLS.

**Figure 6:**
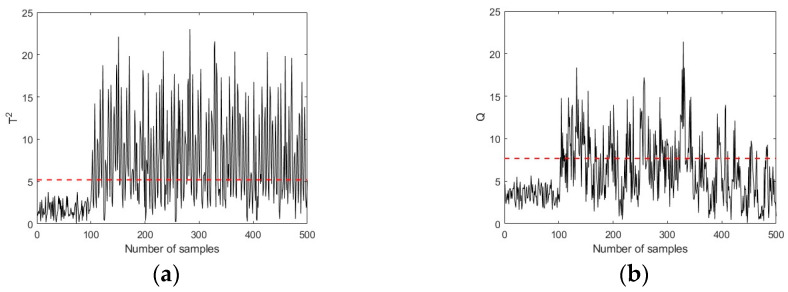
Pump failure detection using PCA (50% full load, 1800 rpm). (**a**) Hotelling’s $T^{2}$ in PCA. (**b**) Hotelling’s Q in PCA.

**Figure 7:**
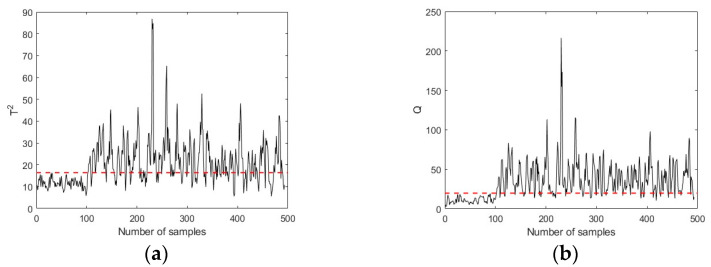
Pump failure detection using CVA (50% full load, 1800 rpm). (**a**) Hotelling’s $T^{2}$ in CVA. (**b**) Hotelling’s Q in CVA.

Figure 5 and Figure 6 shows the fault detection charts using the PLS and PCA-based MSPM approaches, respectively. The first 100 samples are the working data collected during healthy conditions, while samples 101–500 are the fault data. The red lines in Figure 6 represent the UCLs of $T^{2}$ and Q metrics, which are determined based on the training data set. It can be seen that both $T^{2}$ and Q metrics keep below UCLs in the first 100 samples and increase immediately when a fault is introduced (at the 101st time point). The FDR is calculated as 74.75%, which is 38.75% higher than the Pauta criterion, illustrating that the MSPM-based condition monitoring approaches are sensitive to reflect the existence of incipient faults. Figure 7 shows the improvements of the proposed CVA with the AKDE approach for hydraulic system incipient fault detection. Compared with the PLS and PCA-based approaches, the UCLs presented as red dotted lines in Figure 7 fit the fluctuation of the $T^{2}$ and Q metrics better, which means that AKDE can effectively estimate the probability density functions of the $T^{2}$ and Q metrics and assign more accurate upper control limits than the Gaussian assumption approach. In addition, both $T^{2}$ and Q metrics constructed based on the CVA model have good descriptions for incipient faults, showing as a sharp increase in value when a fault is introduced. The fault detection rate of pump failure $f_{1}$ is 86.75%, which is improved by 30% and 12% after using CVA with the AKDE approach, respectively.

The FDRs of all nine fault categories using the Pauta criterion, PLS and PCA-based multivariate statistical approaches and CVA with AKDE are presented in Table 2. The Pauta criterion has unsatisfactory performance for incipient fault detection of the nine fault categories. Most of the FDRs are below 60% except for the filter blockage $f_{3}$ at 25% full load, 1800 r/min and pipe leakage $f_{2}$ at 75% full load, 1800 r/min, for which the FDRs are 70.75% and 64.50%, respectively. This illustrates that the existing univariate statistics-based methods are very limited to detect the hydraulic system’s incipient faults. Multivariate statistical approaches improve the monitoring performance via taking the correlation of various system parameters into consideration. Columns 4 and 5 of Table 2 show the FDRs of the PLS and PCA-based multivariate statistical approaches for the nine fault categories, respectively. Compared with the Pauta criterion, the FDRs of most of the hydraulic lubrication system incipient faults are improved significantly with 41.67% as the maximum increase (pipe leakage $f_{2}$ at 50% full load, 1800 r/min) using PLS and 38.75% as the maximum increase (pump failure $f_{1}$ at 50% full load, 1800 r/min) using PCA. However, for some fault categories, the PLS and PCA-based approach shows unobvious improvements due to the ignoring of the time-varying characteristics of a dynamic system. This shortcoming is well solved by the proposed CVA with AKDE approach. The monitoring results of the marine power plant hydraulic lubrication system are shown in column 5 of Table 2, where the FDRs of all fault categories show a maximum increase of 24.75% (pump failure $f_{1}$ at 75% full load, 1800 r/min). The fault categories that are hard to observed by the Pauta criterion, PLS and PCA-based approaches are well detected by CVA with AKDE. All FDRs of the nine fault categories are larger than 80% with the highest value of 89.25%, which means the proposed approach is effective at detecting different incipient faults under various working conditions.

In order to compare the computation speed and efficiency more intuitively, we use MATLAB to measure the time consumption of PLS, PCA and the proposed method. The computer used is configurated with 32 GB RAM and i5-12500, 3.00 GHz CPU. All the other processes on the computer are closed while testing. For each test, the time consumption is recorded after running the code several times to make the MATLAB run stably. Table 3 shows ten measurements of the time consumption of the PCA and the CVA with AKDE method. Result shows that the PCA takes 1.5096 s to complete the probabilistic reasoning on the average. In contrast, only 1.1678 s is needed with respect to the CVA with AKDE method. The CVA with AKDE method leads to a 20.37% decrease in the time consumption for fault isolation.

## 6. Conclusions

In this paper, a novel nonlinear dynamic hydraulic system incipient fault detection method is proposed by combining CVA with AKDE to overcome the disadvantages of existing approaches on time-independent and Gaussian assumption. The approach is illustrated via a hydraulic lubrication system of a marine power plant. Three common gradual faults are introduced to the system at typical working loads and speeds. The monitoring performances of the proposed CVA with AKDE are compared with the Pauta criterion, PLS and PCA-based MSPM approaches. Results show that the incipient faults that are hard to be detected by the commonly used Pauta criterion can be effectively observed via the MSPM approach. The FDRs of the hydraulic system can be further increased using the CVA with AKDE technique by modelling time-varying characteristics of system states and by directly estimating the underlying PDFs of fault detection metrics, $T^{2}$ and Q. The proposed CVA with AKDE approach remarkably increases the FDRs of hydraulic system incipient faults. The verification of the experimental platform shows that the proposed method can provide an effective method for the condition monitoring and fault diagnosis of dynamic non-Gaussian systems such as hydraulic systems.

## Figures and Tables

**Table 1:** Hydraulic common faults and parameters.

Dataset No.	Fault Type	Working Condition	Dataset Length
A1	pump failure	25% full load, 1800 r/min	400
A2	pump failure	50% full load, 1800 r/min	400
A3	pump failure	75% full load, 1800 r/min	400
A4	pipe leakage	25% full load, 1800 r/min	400
A5	pipe leakage	50% full load, 1800 r/min	400
A6	pipe leakage	75% full load, 1800 r/min	400
A7	filter blockage	25% full load, 1800 r/min	400
A8	filter blockage	50% full load, 1800 r/min	400
A9	filter blockage	75% full load, 1800 r/min	400

**Table 2:** Comparison of different fault detection methods.

Working Condition	Fault Type	Pauta Criterion	PLS	PCA	CVA with AKDE
25% full load, 1800 r/min	pump failure	0.4125	0.7325	0.7250	0.8200
	pipe leakage	0.4025	0.5800	0.6875	0.8250
	filter blockage	0.7075	0.5925	0.7525	0.8400
50% full load, 1800 r/min	pump failure	0.3600	0.6950	0.7475	0.8675
	pipe leakage	0.4125	0.6300	0.7925	0.8925
	filter blockage	0.5675	0.6825	0.7900	0.8750
75% full load, 1800 r/min	pump failure	0.4800	0.6750	0.6400	0.8875
	pipe leakage	0.6450	0.7575	0.7100	0.8225
	filter blockage	0.5700	0.6425	0.6550	0.8725

**Table 3:** The time consumption of PCA and CVA with AKDE.

Method Type	Time Consumption (Second)										
	Test 1	Test 2	Test 3	Test 4	Test 5	Test 6	Test 7	Test 8	Test 9	Test 10	Average
PCA	1.5157	1.5909	1.4964	1.4989	1.4711	1.5309	1.4597	1.5190	1.4909	1.5225	1.5096
CVA with AKDE	1.1756	1.2176	1.2517	1.1536	1.1662	1.1707	1.1116	1.1674	1.1731	1.0909	1.1678

## Data Availability

Not applicable.

## Abbreviations

$x_{k}$	State space vectors
$e_{k}$	Residual space vectors
$u_{k}$	Input vectors
$y_{k}$	Observation vector
f(⋅), h(⋅), g(⋅), and w(⋅)	Unknown nonlinear functions
$p_{k}$	System disturbance noise
$v_{k}$	System measurement noise
A and C	Coefficient matrices
$\varepsilon_{k}$ and $\eta_{k}$	Modelling errors
N	Number of sample data
M	Number of training sets
k	All time points
H	Hankel matrix
$y_{p,k}$	Past column measurement vector
$y_{f,k}$	Future column measurement vector
$Y_{p}$	Past column measurement matrix
$Y_{f}$	Future column measurement matrix
$\rho_{fp}$	Correlation of past and future variables
$a^{T}(y_{f,k})$ and $b^{T}(y_{p,k})$	Coefficient vectors of the linear combinations
U	Left sight singular vectors of Hankel matrix
V	Right singular vectors of Hankel matrix
D	Diagonal matrix of singular values
H	Hankel matrix
$J_{x}$	Projection matrices of state space
F	Projection matrices of residual space
$T^{2}$ and Q	Hotelling’s statistics
$T_{UCL}^{2}$ and $Q_{UCL}$	Upper control limit of Hotelling’s statistics
$h_{0}$	Initial bandwidth
h	Bandwidth
α	Confidence level
$\hat{f}(x)$	Probability density function
$\tau_{i}$	Local bandwidth factor
α	Confidence level
$\hat{f}(x)$	Probability density function
$r_{d}$	Fault detection rate
L	Total number of fault samples
$n_{c}$	Fault sample number identified
$f_{1}$, $f_{2}$, and $f_{3}$	Hydraulic system fault type
$s_{1}$, $s_{2}$, $s_{3}$, and $s_{4}$	Operating parameters of hydraulic system
